# Beyond potency: A proposed lexicon for sensory differentiation of *Cannabis sativa* L. aroma

**DOI:** 10.1371/journal.pone.0335125

**Published:** 2025-10-21

**Authors:** Solomon E. Isaacson, Adrianne R. Wilson-Poe, Tingting Ye, Yanping L. Qian, Thomas H. Shellhammer

**Affiliations:** 1 Department of Food Science and Technology, Oregon State University, Corvallis, Oregon, United States of America; 2 Dow Neurobiology, Legacy Research Institute, Portland, Oregon, United States of America; University of Durham: Durham University, UNITED KINGDOM OF GREAT BRITAIN AND NORTHERN IRELAND

## Abstract

Aroma is a critical factor in consumer-perceived quality of *Cannabis sativa* L., yet standardized tools for describing the aromatic diversity of uncombusted *Cannabis* inflorescence are lacking. This study generated and evaluated a descriptive aroma lexicon for intact *Cannabis* inflorescence consisting of 25 terms with defined reference standards. A human panel evaluated 91 samples using a Check-All-That-Apply method. Multivariate analyses demonstrated the lexicon’s ability to differentiate samples based on orthonasal aroma. Type I and III *Cannabis* exhibited overlapping sensory profiles, though type I (high THC, low CBD) was more frequently described as *skunky*, *musty*, and *animalic*, whereas type III (low THC, high CBD) had higher frequencies of *citrus*, *fruity*, and *candy*-like aromas. Terpene profiling revealed clear chemical clusters, but terpene profiles alone poorly predicted sensory character. Terpinolene was the only compound consistently associated with sensory descriptors, specifically *citrus* and *chemical*. In type III samples, 43 volatile sulfur compounds were detected via gas chromatography with a pulsed flame photometric detector, including dimethyl sulfide, methional, and dimethyl trisulfide while others were tentatively identified or novel. However, neither terpene nor volatile sulfur compound profiles strongly predicted sensory perception. These results emphasize the limitations of chemical composition as a proxy for aroma quality. This work establishes a foundation for future research linking aroma, chemistry, and consumer preferences, and supports the development of quality metrics beyond delta-9-tetrahydrocannabinol potency.

## Introduction

Cannabis and hemp are both classified botanically as *Cannabis sativa* L., a single species within the Cannabaceae family. In the United States, the distinction between them is legally defined based on the concentration of delta-9-tetrahydrocannabinol (THC), with hemp containing 0.3% THC or less by dry weight, whereas plant material exceeding this threshold is classified as drug-type cannabis, despite the absence of fundamental taxonomic or morphological differentiation [1,2]. Due to the arbitrary nature of this distinction *Cannabis* is scientifically classified by chemotype, which is based on the ratio of cannabinoids within the plant. *Cannabis* which is THC-dominant is considered “type I”, balanced THC:CBD is “type II”, CBD-dominant is “type III”, CBG-dominant is “type IV” and with little to no cannabinoids is “type V” [3].

*Cannabis* has been used globally as a food, fiber, and medicinal commodity for millennia [4]. In the United States, federal regulation of the plant began in 1937 with the passage of the Marihuana Tax Act, followed by its complete removal from the National Formulary and Pharmacopoeia in 1941 [5]. In 1970, the Controlled Substances Act passed under the Nixon administration criminalized production and possession of the plant, which restricted the ability to conduct research on *Cannabis* [5]. These restrictions have impeded formal research on *Cannabis* in the modern era, limiting the development of a comprehensive understanding of its defining characteristics, as has been achieved with hops, coffee, winegrapes, and other agricultural commodities [6].

A critical knowledge gap exists in establishing a consensus on physical and chemical features that determine the quality of *Cannabis* inflorescence. Previous studies have found that consumers differentiate type I *Cannabis* products on the basis of perceived quality, yet the difference between “low- mid- and high-grade” *Cannabis* remains undefined [7]. Decades of illicit drug market economics have falsely led consumers in legal *Cannabis* markets to conceptualize THC potency as a marker of quality, despite the fact that neither THC potency nor dose correlates with pleasant subjective effects [8]. In the absence of scientific guidance on quality, consumers have turned to potency as a guiding feature for purchasing [9]. Multiple studies have shown that in regulated markets, prices of *Cannabis* are positively correlated with THC concentration [2,10]. Consumers associate increased price with increased quality, pushing quality-conscious consumers towards products with higher and higher THC content. Because THC poses dose-dependent health risks such as driving impairment, cannabinoid hyperemesis, and acute psychosis, the conflation of perceived quality with THC potency poses significant public health risks, and novel markers of product quality are warranted [11]. A recent large-scale observational study sought to address this knowledge gap by defining product quality based on the ability to elicit pleasant subjective effects, the results of which suggest that, similar to coffee and tea, pleasant aroma is the key criterion defining high quality *Cannabis* inflorescence [8]. This finding are corroborated by previous studies in this field, which have shown that scent impacts consumer perception of quality in *Cannabis* [12].

Although previous work highlights the importance of aroma as a criterion of product quality and consumer demand, there is a paucity of rigorous evidence defining the chemical characteristics of *Cannabis* aroma in whole inflorescence prior to consumption or aerosolization. While much attention has been placed on terpene composition, recent literature shows a broad range of compounds beyond terpenes may play a critical role in the aroma of *Cannabis*. Growing evidence suggests that esters, aldehydes, thiols, and other volatile organic compounds significantly influence the sensory profile of *Cannabis* inflorescence [13–16]. Many esters and aldehydes have been identified in *Cannabis*, however there is little work that links their presence to specific aromas and what does exist focuses on extracts rather than whole inflorescence. Tropical fruit-smelling volatile sulfur compounds (VSCs) such as thiols and prenylated sulfur compounds have previously been identified in type I *Cannabis* extracts [14]. These highly potent VSCs, such as 3-sulfanylhexanol, 3-sulfanylhexyl acetate, 3-sulfanylhexyl butyrate, have also been identified in other foods and are known to be responsible for fruity aromas such as guava, passionfruit, or black currant, respectively [17,18]. Prenylated compounds, such as 3-methyl-2-butene-1-thiol, or 3-methylthiophene have been shown to contribute skunky, savory notes to type I *Cannabis* extracts [13]. As a result of this growing body of research, this study included an examination of the VSCs present in type III *Cannabis* samples.

Most existing *Cannabis* aroma taxonomy efforts have focused solely on type I *Cannabis*, and their application is limited given that industry-funded studies were not conducted in unbiased research settings, nor did they rely on trained sensory analysts [12,19–21]. The need for a clear descriptive aroma lexicon for *Cannabis* inflorescence is growing. A standardized, consistently applied descriptive lexicon for *Cannabis* would facilitate consistent communication among cultivators, product developers, distributors, retailers, and consumers. Furthermore, this research could contribute to a broader definition of product quality that goes beyond the concentration of psychoactive compounds [22]. Because aroma is independent of THC potency and its associated health risks, understanding which aromas are most important to consumers and establishing consistent definitions for these aromas is essential to public health [12]. While consumer hedonics, which are crucial to define quality, are not typically included in lexicon development, the work presented herein moves the field in the direction of having consistent, standardized language for describing *Cannabis* aroma [23,24].

The objective of this study was to generate a standardized aroma lexicon for characterizing the aromatic attributes of *Cannabis* inflorescence with an associated sensory method. Given the morphological, genetic, and phytochemical similarities between type I and III *Cannabis* varieties commercially available in state-legal markets, a secondary aim of this study was the exploration of the hypothesis that these products share overlapping sensory profiles. Additionally, we aimed to assess the variability in terpene, terpenoid, and VSC composition among *Cannabis* samples and to evaluate potential associations between chemical constituents of the essential oil fraction and sensory attributes.

## Methods

### *Cannabis* selection

A total of 59 type III *Cannabis* samples were collected from five commercial growers in in two countries: 49 from the United States (30 from Oregon, 9 from California, 10 from Washington) and 10 from Switzerland. All US samples underwent pre-harvest testing to comply with the U.S. Farm Bill and were exempt from Schedule I drug classification. All state and federal regulations governing importation and chain of custody were strictly adhered to for samples sourced from Switzerland, California, and Washington. The 30 Oregon samples were sourced from two Oregon Department of Agriculture-certified commercial cultivators, Horn Creek Hemp and East Fork Cultivars. Horn Creek provided 18 samples, all of which were barn-dried using conventional commercial practices; three of these were hand-trimmed at Oregon State University (OSU), while the remaining samples were machine-trimmed by the cultivator. East Fork provided 12 barn-dried, hand-trimmed samples. In northern California (Sebastopol), study investigators collected nine samples from an experimental plot within a commercial operation, who opted to remain anonymous. A commercial grower in Washington (Vuca Farms, Yakima, WA) provided an additional 10 samples. All samples from California and Washington were freshly harvested by the study team and immediately transported by refrigerated truck to OSU, where they were dried and processed (see below). A set of 10 samples were provided by Puregene Ag, a commercial cultivator in Switzerland. All Swiss samples were hand-trimmed, six of which were barn-dried, two were freeze-dried, and two were dried using forced air. All dried and trimmed samples were stored at 4°C in high-barrier flexible pouches flushed with nitrogen. For each type III cultivar in the study, on average 700 grams of dried inflorescence was obtained (ranging from 300–1000 g).

To compare the aromatic characteristics of commercial type III samples with those of commercial type I, a total of 32 type I samples from 15 different cultivators were curated by a licensed retailer, High Quality LLC in Corvallis, OR. Sample selection by *Cannabis* industry professionals ensured a diverse and market-representative set. Although an equal number of type I and III samples would have been ideal, it was not feasible to procure 59 unique type I samples at the time of study. Importantly, because the primary objective of this work was lexicon development and product differentiation rather than direct statistical comparison, the uneven sample numbers do not compromise the validity of the findings. All type I samples were produced by Oregon Liquor and Cannabis Commission (OLCC)-licensed producers within the state of Oregon, laboratory tested for purity and potency and compliantly routed to the retailer. All type I samples always remained in the custody and control of the licensed retailer. Type I samples were stored in high-barrier flexible pouches under ambient room temperature conditions prior to testing. For each type I cultivar, approximately 14 – 28g was sequestered.

### Green inflorescence handling and processing at Oregon State University

Type III samples from Washington were harvested from Vuca Farms on October 12, 2023, and type III samples from California were harvested on October 26, 2023. To preserve volatile compounds, immediately after harvesting, samples were transported to Corvallis, Oregon via refrigerated truck. Stalks of plants with inflorescences attached were hung on support structures and dried under forced-air conditions using the Multi-Chamber Modular Environmental Conditioning System (MCMEC) at OSU. The chamber was initially set to 15.5°C and 20% relative humidity (RH) until the samples reached a water activity below 0.8 to prevent mold growth, a process that took approximately 3.5 days. The chamber’s conditions were then adjusted to 60% RH, and the samples were allowed to dry until their moisture content reached approximately 10%, which took an additional 9–10 days (S1 Fig). This drying technique was selected to preserve volatile aromatic compounds as much as possible. Following drying, the inflorescences were removed from the stalks and hand trimmed to remove unwanted leaf material. The dried inflorescences were placed in high-barrier flexible pouches and stored at 4°C until analysis.

### Sensory analysis

#### Lexicon development.

The lexicon development for this study began with post-hoc mining of open-ended archival comments from judges in the 2020 Cultivation Classic, a commercial *Cannabis* competition held in Portland, OR which was initially reported on in Plumb et al. 2023 [8]. It included aromatic descriptors from 196 regular *Cannabis* consumers (untrained in sensory analysis) who evaluated a total of 134 *Cannabis* samples, with each volunteer evaluating 8–10 1-gram samples. Volunteers were provided with an open text field and asked to choose one to three words to describe the aroma of the samples. The complete open text field dataset, containing a total of 4,325 descriptor instances, was generously provided for this study. 81 unique descriptors appeared in this data set, and those that appeared most frequently across the samples were compiled and refined from 81 to 25 descriptors.

This process provided a preliminary version of the lexicon, which was further refined by comparing with language published by commercial vendors of *Cannabis* terpenes products, such as True Terpenes® and Abstrax Tech [25,26]. The lexicon was further refined through one-on-one discussions with industry professionals, such as a retail intake manager and a senior staff member at a commercial *Cannabis* dispensary in Corvallis, several commercial type III cultivators who provided samples for this study, and additional type III *Cannabis* growers and breeders within the OSU Global Hemp Innovation Center, who were not involved with the study.

Finally, a bench trial was conducted with the study team plus five sensory-trained laboratory members and two *Cannabis* industry professionals, to assess the completeness of the ballot with a preliminary selection of the type III cultivars in the study selected from the larger experimental set. Feedback from the bench trial was used to refine and finalize the lexicon used in all subsequent sensory analysis sessions (Table 1).

**Table 1:** Sensory lexicon with definitions and aroma standard compositions.

Descriptor	Definition	Aroma Standard Composition
Fruity	Fruit cocktail (pear, peach, grape)	Canned fruit cocktail in heavy syrup (Kroger)
Citrus	Aromas of citrus and citrus peel: lemon, lime, orange, grapefruit, etc.	Grapefruit and lemon peel
Berry	Aromas of blueberry, strawberry, raspberry, etc.	Triple berry medley (Private Selection)
Tropical	Aromas of tropical fruit such as pineapple, mango, etc.	Passion orange guava juice (Sun Tropics)
Earthy	Damp forest duff or freshly dug moist earth	0.01% w/w Geosmin in propylene glycol (Perfumer’s Supply House)
Musty	Damp basement, dusty aromas	5 PPB 2,4,6 – Trichloroanisole in propylene glycol (Sigma Aldrich)
Woody	Aromas of pine, cedar, etc.	Cedar Oil (NOW Essential Oils)
Black Tea	Black tea aroma	Black tea (Lipton)
Pepper	Aromas of fresh cracked black pepper	Fresh cracked black pepper (McCormick)
Herbal	Aromas of sage, mint, camphor, menthol, etc.	Rubbed sage (McCormick)
Floral	Aromas of violet, jasmine, rose, etc.	0.03% Methyl jasmonate in propylene glycol (Perfumer’s Supply House)
Straw	Dried grass or hay	Dry barley straw
Baking spices	Aromas of cinnamon, clove, nutmeg	Whole cloves with ground cinnamon (McCormick)
Nutty/ Toasted bread	Toasted nut and/or white bread	Toasted white bread (Kroger Classic White)
Skunky	Natural gas, burnt rubber	0.01% 3-methylbutanethiol in propylene glycol (Toronto Research Chemical)
Fuel	Diesel and gasoline aromas	N/A
Chemical	Wet glue, polish remover, plastic, rubber aromas	N/A
Ammonia	Ammonia, cleaning solution	0.15% Ammonia in propylene glycol (Kroger)
Cheesy	Aromas of hard cheeses	Parmesan cheese (Boar’s Head)
Creamy	Aromas of sweet dairy, cheesecake	Heavy whipping cream with 5% w/w vanilla extract added (Kroger)
Candy	Aromas of Pez/Skittles®/SweeTarts candy	2% Maltol in propylene glycol (Perfumer’s Supply House)
Cakey	Aromas of vanilla, brown sugar, sugar cookie, cakey, wedding cake, honey	Hydrated white cake mix (Betty Crocker)
Doughy/Yeasty	Fermenting bread dough	Pizza dough
Animal	Barn farm, horse blanket aromas	0.5% w/w 4-ethyl phenol in propylene glycol (Sigma Aldrich)
Vomit/fecal	Vomit or feces	N/A

#### Aroma standard development.

For 22 of the 25 aroma terms in the lexicon, external aroma reference standards were developed. The aroma terms *fuel*, *chemical*, and *vomit/fecal* did not have external reference standards due to safety concerns associated with potential compounds representing these odors, and because these descriptors are generally familiar to most panelists without additional training. Unlike nuanced terms such as *citrus*, which require calibration, these odors are more easily recognized. Using an iterative approach, the concentrations of individual compounds or sources of food standards were adjusted until they clearly represented the terms in the ballot (Table 1).

When chemical standards were used, they were purchased as pure standards except for 3-methylbutanethiol (3-MBT) which was prepared using an enzyme kit supplied by Toronto Research Chemicals (Toronto, Canada). For standards prepared in propylene glycol, 0.5mL was placed into a 60 ml lidded plastic black ramekin, along with a small piece of filter paper to absorb the liquid and reduce the possibility of spills. For the food standards, the ramekins were filled approximately halfway with the standard or until there was enough that it could easily be smelled once the lid was removed. Aroma standards were prepared the same day training was conducted to ensure they were fresh.

#### Recruitment and panelist training.

Panelists were recruited from the Corvallis community using existing panelist pools from ongoing food and beverage sensory evaluation panels at OSU as well as in-person recruiting from local *Cannabis* retailers in Corvallis. Panelist recruitment was conducted from July 11^th^, 2024 to July 23^rd^, 2024. Exclusion criteria for panelists included whether they found the aroma of *Cannabis* offensive, were allergic to *Cannabis*, viewed *Cannabis* use as immoral, had taste or smell deficits, had experienced *Cannabis* use disorder, or were under the age of 21. The panelist recruitment and sensory data collection was carried out under a research protocol approved by the Oregon State University Institutional Review Board (#HE-2023–730). Written consent was obtained from each participant prior to any training or data collection.

The sensory descriptive panel for type III *Cannabis* analysis consisted of 24 panelists (10 male, 14 female) aged 21–70 years, with a median age of 31.5. Eighteen panelists had prior experience with descriptive analysis of food and beverages. Panelists were screened for their familiarity with type I *Cannabis* aroma and classified as slightly familiar (six panelists), moderately familiar (ten panelists), or extremely familiar (eight panelists). For the type I analysis, the panel was reduced to 21 panelists (9 male, 12 female), aged 21–70, with a median age of 32. Among them, five were slightly familiar, nine were moderately familiar, and seven were extremely familiar with *Cannabis* aroma. Previous research has shown that familiarity with a product category can enhance panelists’ ability to discriminate among sensory attributes [27].

Before sensory data collection began, each panelist participated in training sessions to familiarize themselves with the ballot and aroma standards (Table 1). Training sessions were conducted as open-ended discussions in groups of two to nine panelists, where panelists were led through smelling aroma standards by the study lead with time to discuss them and clarify any potential confusion. At the end of each training session, panelists smelled three type III samples as a group and came to a consensus on which attributes they felt applied to validate the training. Each training session lasted approximately 45–60 minutes and was conducted by the same panel leader with the standards and samples presented in the same order. Each panelist was required to participate in two training sessions to ensure familiarity with the standards and ballot.

#### Sensory data collection.

Evaluation of 59 type III samples was conducted by all 24 panelists at OSU in a room routinely used for sensory data collection. To ensure legal and state-compliant handling of type I samples, the evaluation of those 32 samples was not performed at OSU but was carried out at an indoor event space adjacent to, owned by, and under the control of a local *Cannabis* dispensary (High Quality LLC). Only 21 of the original 24 panelists participated due to scheduling conflicts. Evaluations in both locations were executed identically using a within-subjects design. All evaluations took place in well-lit rooms with minimal decoration or background odors. Panelists were not allowed to converse with others within the testing room during data collection.

Each evaluation session lasted approximately 30–60 minutes and involved evaluating 9–11 samples, all of which were prepared on the day of the evaluation. Samples consisted of approximately one gram of intact, dried inflorescence, presented in lidded 60 ml black plastic ramekins labelled with randomized three-digit codes. Panelists evaluated the aroma of the inflorescences and recorded their impressions before moving on to the next sample. Panelists did not smoke or consume the samples as part of their evaluation. Sensory data collection was conducted using a Check-All-That-Apply (CATA) ballot built using Qualtrics survey software and accessed via tablet computers provided by the investigators. For each sample and panelist, the terms in the ballot were presented in a randomized order. Additionally, while each session contained the same randomly selected samples for all panelists, samples were assessed in a panelist-specific random order. Panelists were allowed as much time as they wanted to evaluate each sample, with a forced 60 second break between samples. The sensory data collection consisted of seven type III evaluation sessions followed by three additional type I evaluation sessions, resulting in a total of twelve sessions which were performed over a three-week period.

### Chemical analyses

#### Terpenes and terpenoids.

Terpene analysis was performed by a commercial lab that routinely quantifies cannabinoid and terpene composition of *Cannabis* (Columbia Labs, Portland, OR) using an industry standard method [28]. Samples were tested for the presence of 40 terpenes (S1 Table). Terpene extraction was carried out by placing a 0.2 g sample of *Cannabis* inflorescence into a centrifuge tube to which 10 mL hexane was added before mixing on an oscillating shaker at 600 OSC/min for 5 minutes. Samples were centrifuged at 3500 RPM for 10 minutes and 1 mL of supernatant was placed into amber GC sample vials and stored in a freezer until chromatographic analysis.

A one (1) μL sample of the hexane extract was injected in split mode on an Agilent 8890A gas chromatograph (Agilent Technologies, Inc., Santa Clara, CA, USA) using a split ratio of 20:1 with the injector maintained at 250°C. Compounds were separated with a Restek Rxi-5Sil MS 30 m x 0.25 mm ID x 0.25 um film w/ 5 m guard (Cat. 13623−124). The glass splitter led to 0.3 m x 0.25 mm ID (FID) and 0.8 m x 0.1 mm (MS) restrictor columns. The carrier gas was hydrogen set to a flow rate of 4.5 mL/min. The oven ramp rate was 2°C/min until 50°C was reached, 6°C/min until 115°C was reached, 15°C/min until 160°C was reached, and then 30°C/min until 330°C was reached (total run time of 25 minutes). Both Flame Ionization Detector (FID) and Mass Spectrometer (Agilent 5977B MSD) were used for detection. The FID was set to 290°C, with hydrogen flow of 35mL/min, nitrogen flow of 10 mL/min and air flow of 350 mL/min. The data collection rate was 50 hertz. The MS had a solvent delay of 5.00 min and scan speed of N = 4. The low mass was 35 amu and high mass was 260 amu, with a threshold of 100 amu. The software used to process the resulting data was Agilent Mass Hunter v10.2. Commercial certified multi-component terpene mixtures were used as analytical standards for quantitation using a standard curve (Terpene Mixtures 1 and 2, LGC Group, Middlesex, UK).

#### Volatile sulfur compounds.

Analysis of VSCs was carried out at OSU using HS-SPME-GC coupled with a pulsed flame photometric detector (PFPD). This detector was chosen due to its specificity for sulfur compounds. The analysis was only run on type III samples to avoid DEA licensing and the complications of having high-THC samples on campus.

Approximately ten grams of each sample was separated and stored at −10 °C in sealed high-barrier bags flushed with nitrogen. On the day of analysis, 1 g of inflorescence was weighed into a vial with 5 mL 5% ethanol in deionized water and shaken for 30 minutes at 250 rpm. 10 µL of 2.5 ppm diisopropyl disulfide (DIDS) was added as an internal standard after shaking. Each sample was prepared in quadruplicate.

The samples were then incubated and agitated at 50°C and 500 rpm for 5 minutes before headspace analysis with a 2 cm SPME fiber (DVB/CAR/PDMS, divinylbenzene/carboxen/polydimethylsiloxane, 50/30 µm film thickness, Supelco, Bellefonte, PA, USA). The fiber was left in contact with the headspace for 20 minutes, during which time the temperature was maintained at 50°C and stirring was continued at 250 rpm. Compounds were desorbed from the fiber for 5 min 30 seconds at 300°C in splitless mode in the GC (Varian CP-3800 Gas Chromatograph, Varian, Inc., Walnut Creek, CA, USA) injection port. The injector was a 1079 split/splitless injector (Varian, Inc., Walnut Creek, CA, USA), and the extraction and injection were conducted in CTC Analytics Combi PAL Autosampler (Agilent Technologies, Inc.). Compounds were separated using a DB-FFAP column (30 m × 0.32 mm I.D.,1 µm film thickness, Agilent Technologies, Inc.), and the oven ramp rates were as follows: 35°C held for 3 min, then ramped at a rate of 10°C/min to 150°C and held for 5 min, followed by a 20°C/min ramp rate to 220°C and held for 3 min. Nitrogen was used as a carrier gas with a flow rate of 2 mL/min. The PFPD was set to 300°C, and gasses were provided as: air1 = 17 mL/min, H2 = 14 mL/min, air2 = 10 mL/min.

Analysis was carried out using Star Chromatography Workstation Version 6 (Varian, Inc.), using retention times to tentatively identify compounds. The square root ratio of the peak area of each sulfur compound was calculated to account for the exponential response of the detector. Each sample was prepared in quadruplicate. External standards were run for 3-sulfanyl-1-hexanol (3-SH), 3-sulfanyl hexyl acetate (3-SHA), 3-methyl-2-butene-1-thiol (3-MBT), ethanethiol, 3-methylthiophene, and 2-mercaptoethanol.

Analysis of dimethylsulfide (DMS) was carried out separately from other VSCs using HS-GC-PFPD. 1g of inflorescence was weighed into a vial with 5 mL 5% ethanol in deionized water and shaken for 30 minutes at 250 rpm, to which 10 µL of 200 ppm ethyl methyl sulfide (EMS) was added as an internal standard. Each sample was prepared in triplicate.

Before injection, each sample was incubated at 45°C for 20 min with agitation. 1 mL of the headspace syringe maintained at 50°C was used for headspace sampling while stirring at 500 rpm. 0.5 mL was injected in splitless mode, with the temperature of the injection port set 250°C. The column, oven ramp, and PFPD configuration were the same as above. A seven-point calibration curve was prepared for DMS to allow quantitation. The square root ratio of the peak area of DMS to the internal standard was used for calculation.

### Statistical analysis

The data collected through the CATA ballot was analyzed as an aggregate of frequency across both sample typers. After sensory data was collected, the frequencies of responses were tabulated to form contingency tables for all attributes and all samples. Only data from the 21 panelists who participated in both type I and III sensory sessions was considered for most analyses performed, the exception being for the type III-only analyses which included the full 24 panelist data set. All statistical analyses were performed in XLStat (XLStat Sensory, Version 2024.4.0, Addinsoft), including analysis of variance (ANOVA), principal component analysis (PCA), correspondence analysis (CA), multi-factor analysis (MFA), and partial least squares regression (PLSR). Preprocessing of the chemistry data via mean centering and scaling by standard deviation was performed prior to PLSR analysis.

## Results

### Panelist performance

The CATA ballot in this study resulted in 8,075 descriptor instances used across all 91 samples and 21 judges. Agglomerative hierarchical clustering (AHC) was used to group samples based on sensory similarity. The sample set included four functional duplicates from the same producer and production lot, with half of the biomass machine-trimmed at the farm, and the other half hand-trimmed at OSU by study investigators. AHC revealed that these four pairs of duplicates clustered together as neighbors, indicating a high degree of sensory similarity (S2 Fig). These results indicate that the panel as a whole was able to rate duplicate samples almost identically which allows us to infer a high degree of repeatability in the panel.

### Lexicon assessment

A total of 2,834 (35%) descriptor instances were recorded for the 32 type I samples and 5,241 (65%) were provided for 59 type III samples. On average, panelists used nearly the same number of descriptor instances for both sample types (88.8 per type III sample and 88.6 per type I sample), indicating consistent engagement with the ballot across sample types and testing locations.

Although previous reports utilized larger descriptive lexicons for *Cannabis*, ranging from 48 to 82 descriptors [12,21], the 25-descriptor lexicon used in this study was sufficient to allow panelists to successfully differentiate samples. Cochran’s Q test was performed on the data resulting from CATA analysis for both combined samples (n = 21 panelists) and type III-only (n = 24 panelists) to test for significant (p ≤ 0.05) sample effects across the descriptive attributes. The panel was able to find significant differences among the samples for all attributes, except for *baking spice*, which approached statistical significance (p = 0.063). In a sub analysis exclusively of type III samples, *floral* was the only term which failed to reach significance (p = 0.110). These results point to the efficacy of nearly all descriptive attributes in discriminating samples.

The terms *herbal*, *citrus*, and *woody* were used most frequently, accounting for 26% (2,083) of the 8,075 total descriptor instances. *Herbal* was identified in all samples, and in 82 out of 91 samples (90%), it was reported by more than five panelists. Compared to type I samples (average frequency of 7.9), *herbal* was reported more frequently in type III samples (9.4) (S2 Table). Because it was used at high levels across all samples, *herbal* was less effective than other descriptors in differentiating samples. It distinguished samples with low frequencies of this attribute more effectively than those with high frequencies.

*Citrus* was the second most frequently used descriptor, but in contrast to *herbal*, there was greater variation in its frequencies of use. On average, *citrus* was also reported more frequently in type III (8.1) than in type I samples (5.8). At least five (out of 21) panelists utilized the *citrus* descriptor in just 55% of the samples, yet it accounted for 8% (662) of the total frequency count. This suggests that *citrus* was a key differentiating feature. Unlike *herbal*, which was present at high frequencies in most samples, *citrus* had considerably more variation in frequency of use. The use of *citrus* as a descriptor was more binary in nature than other descriptors. That is, if it was detected, most panelists identified it indicating it was an important defining attribute for those samples where it was present.

The third most used descriptor was *woody*. At least five panelists utilized the descriptor *woody* in 70% of the samples, and it accounted for 8% (615) of the total descriptor instances. Most samples had middling amounts of *woody* rankings and were not easy to distinguish, however samples with very low or very high levels of *woody* notes were more easily differentiated. *Woody* was used equivalently across sample types, with average frequencies of 6.8 in both categories.

### Type I and III *Cannabis* share a similar sensory space

**Fig 1:**
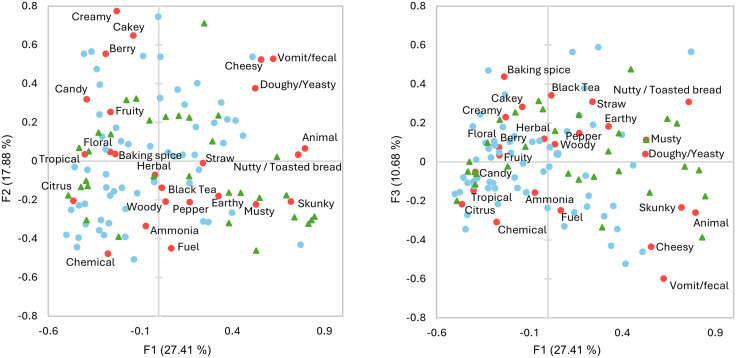
Correspondence analysis biplot of type I and type III sensory spaces with type I shown in green, type III shown in blue, and sensory attributes shown in red.

**Fig 2:**
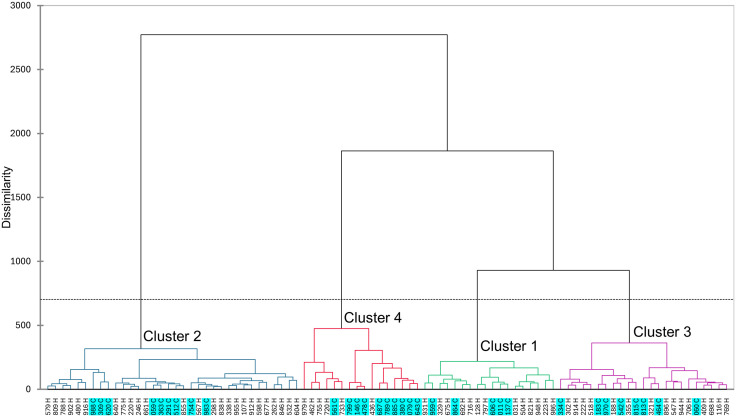
Agglomerative Hierarchical Clustering of type III and type I (blue highlight) samples based on sensory attributes.

Type I and III *Cannabis* share a similar sensory space as demonstrated by the generally even distribution of the samples through most sensory clusters (Table 2), the relatively similar data dispersion in the CA biplot (Fig 1), and the small differences in average attribute frequencies between sample types (S2 Table). Nonetheless, there were some important differentiating features, namely *herbal*, *citrus*, *fruity*, *candy*, *floral*, and *tropical* were more frequently selected for type III samples while *ammonia*, *musty*, *fuel*, *animal*, *skunky*, and *nutty/toasted bread* where more frequently used for type I samples (S2 Table). AHC was used to generate a dissimilarity dendrogram based on aroma descriptors, facilitating further comparisons of sample types (Fig 2 and Table 2). Clusters 1 and 2 had slightly more type I than III samples, and cluster 3 had both types at approximately the same rate as their inclusion in the study (Table 2). Cluster 4 was substantially smaller than other clusters and contained almost twice as many type I sample than type III. While all four clusters had high frequencies of *herbal*, Cluster 1 was differentiated from the others by very high frequencies of *fruity*, *berry*, *candy*, and *cakey*, Cluster 2 by *citrus* and *chemical*, Cluster 3 by *cheesy* and *vomit/fecal* and Cluster 4 (highest in type I samples) by *earthy, musty*, *straw*, *black tea*, *skunky*, *nutty/toasted bread*, and *woody* (S3 Table and S3 Fig). Although CATA is not a direct measure of sensory intensity, the frequency of attribute selection in this study provides a strong reflection of the relative intensities perceived for these attributes, a relationship that has also been documented by the originator of the CATA method [29].

**Table 2:** Results of Agglomerative Hierarchical Clustering on type I and III samples based on chemistry and sensory results.

	Terpene				Sensory			
**Cluster**	A	B	C	D	1	2	3	4
**Size**	39	18	18	16	18	34	23	16
**Type III**	35	11	2	11	13	25	15	6
**Type I**	4	7	16	5	5	9	8	10
**Type III**	90%	61%	11%	69%	72%	74%	65%	38%
**Type I**	10%	39%	89%	31%	28%	26%	35%	63%

### Terpene analysis

**Fig 3:**
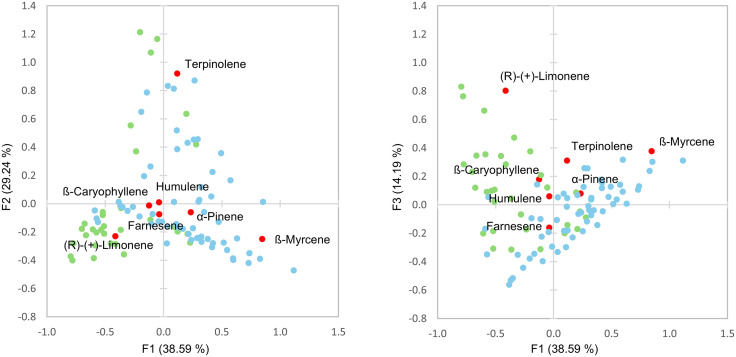
Principal Component Analysis biplot of type III (blue) and type I (green) samples based on terpene chemistry (red).

PCA was performed on the terpene data to identify the important terpenes for assessing variation among and within the samples (Fig 3). Of the 21 terpenes found above the limit of quantitation in any sample, seven accounted for most of the variation: ß-myrcene, d-limonene, terpinolene, α-pinene, humulene, beta-caryophyllene, and farnesene (Table 3). Of these, just three compounds (myrcene, terpinolene, and limonene, in that order) were responsible for most of the variation explained by the first three components of the PCA and representing over 80% of the terpene variation in the data set.

**Table 3:** Contribution of terpenes to Principal Component Analysis biplot of terpene chemistry.

	F1	F2	F3	F4	F5
% Variation	38.6	29.2	14.2	7.2	5.2
Humulene	0.1	0	0.3	6.1	8.4
α-Pinene	5.5	0.4	0.6	0	3.6
Terpinolene	1.4	84.6	9.6	0.7	1.3
ß-Myrcene	71.5	6.3	14.2	0.6	0.6
ß-Caryophyllene	1.5	0	3.2	33.5	43.2
(R)-(+)-Limonene	17.1	5.3	64.4	0.2	4.7
Farnesene	0.1	0.6	2.6	54.2	35.3

AHC was used to separate samples into four clusters based on terpene chemistry data (Table 2). Clusters B and D contained samples at rates similar to their inclusion in the study, about 65% type III and 35% type I. However, Cluster A was comprised of 90% type III samples and Cluster C was comprised of 89% type I samples. Least Square (LS) Means comparisons of the samples was performed to find significant differences in terpene concentrations between the clusters (S4 Table). Cluster A (type I dominant) was differentiated by high concentrations of ß-myrcene and α-pinene. Cluster B was differentiated by high concentrations of terpinolene, d-3-carene, α-terpinene, trans-ß-ocimene, and α-phellandrene. Cluster C (type III dominant) was differentiated by higher concentrations of fenchol, α-terpineol, linalool, humulene, ß-caryophyllene, and especially d-limonene. Finally, cluster D was differentiated by high concentrations of guaiol, and farnesene, and low concentrations of most other terpenes.

### Volatile sulfur compound analysis

**Fig 4:**
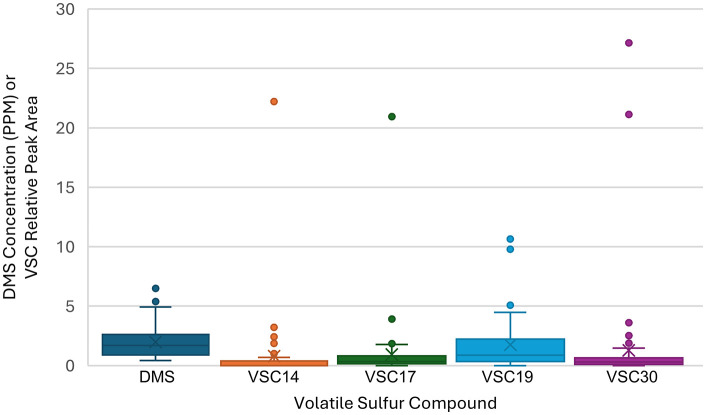
Box and Whisker plot of DMS concentration (PPM) and relative peak areas of VSCs 14, 17, 19, and 30.

Type III samples in this study were analyzed using gas chromatography coupled to a PFPD, which is selective for sulfur-containing compounds thereby enabling detection of VSCs. Without having a broad set of existing chemical reference standards, many observed chromatographic peaks could not be confidently identified. Among the identified compounds, DMS was the only one robustly quantified. The mean concentration of DMS was 1.98 ± 1.35 ppm, with a range from 0.42 ppm to 6.50 ppm (Fig 4).

A total of 43 sulfur-containing peaks were detected across 59 samples. Compounds identified with certainty were methional, DMS, hydrogen sulfide (H₂S), diethyl disulfide (DEDS), methanethiol (MeSH), methyl thioacetate (MeSOAc), ethyl thioacetate (EtSOAc), and dimethyl trisulfide (DMTS). While DMS has been identified in prior literature [13], the other compounds appear to be novel within the context of *Cannabis* inflorescence. Reference standards for 3SH, 3SHA, and 3MBT were also analyzed. Of these, 3MBT was of particular interest due to its reported role in driving skunky aromas in type I *Cannabis* [13]. A tentative match was observed between sample peaks and the 3MBT standard, however definitive identification was not possible due to the presence of impurities in the 3MBT standard. We suspect the impurities came from the standard preparation via an enzyme kit. 3SH and 3SHA were not identified in any samples.

**Fig 5:**
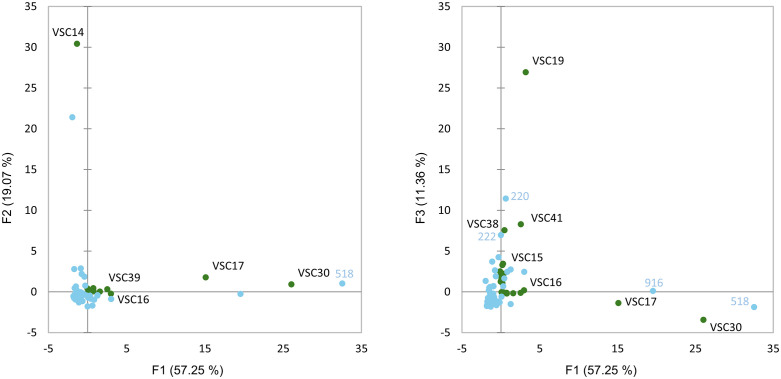
Principal component analysis biplot of type III samples (blue) based on VSC (green) chemistry.

Compared to the terpenes analyzed in this study, the overall variability in VSC concentrations across the sample set was considerably lower (Fig 5). The samples appeared quite similar, with a small number of VSCs accounting for a vast majority of variation. VSCs 14, 17, 19 and 30 were shown to account for most of the variation within the type III samples, with four samples (220, 518, 797, and 916) having very high concentrations of those compounds relative to the rest of the sample set. Interestingly, the retention times of VSCs 17 and 30 aligned with peaks in the 3MBT standard, indicating that one of them was potentially this compound.

**Fig 6:**
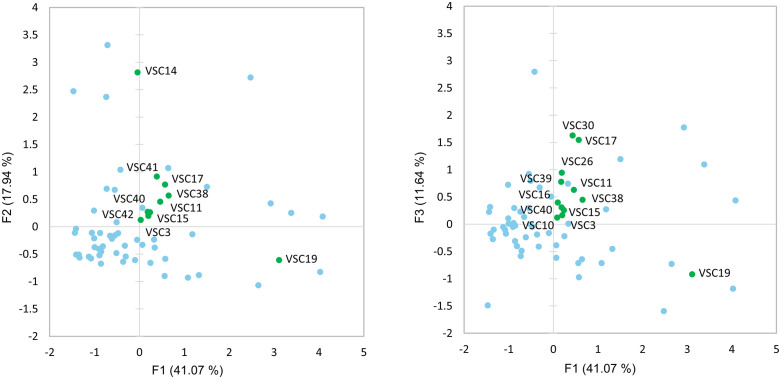
Principal component analysis of VSC data with outliers removed.

To better assess VSC covariation within the bulk of the type III samples, the four extreme samples noted earlier were removed from the dataset and then reanalyzed via PCA (Fig 6). Even after their removal, VSCs 14, 17, 19, and 30 still accounted for most of the variation within the samples. Future work would benefit from identifying these compounds.

### Challenges linking chemistry to sensory attributes

Despite statistically significant differences in terpene content among sensory-defined clusters (S5 Table), the ability of terpene chemistry to serve as a reliable predictor of sensory characteristics in *Cannabis* was limited. Among the suite of terpenes analyzed, terpinolene emerged as the most salient compound, demonstrating a notable association with both *citrus* and *chemical* aroma attributes. Specifically, sensory cluster 2, which was rated more than twice as high as any other cluster for these two descriptors, exhibited a terpinolene concentration nearly eightfold greater than the sample mean (S5 Table). However, beyond this singular association, few terpenes showed consistent or robust predictive relationships with sensory qualities.

Cluster 4 offered an instructive counterexample: characterized by high intensities of *earthy*, *musty*, *straw*, *black tea*, *skunky*, *nutty/toasted bread*, and *woody* notes, it exhibited elevated levels of ß-caryophyllene, linalool, humulene, and an especially high concentration of d-limonene. This profile contradicts the prevailing assumption in popular culture and scientific literature that d-limonene is a key driver of citrus aroma in *Cannabis*, as this cluster lacked strong citrus sensory attributes despite its high d-limonene content [30–33]. This lack of association is not entirely surprising given the capacity for the quality of an odorant within a mixture to change depending on the other odorants present [34].

Exploratory multivariate analyses further underscored the weak correspondence between terpene chemistry and sensory perception. The PCA of terpene profiles failed to generate coherent groupings that corresponded to sensory-defined clusters (S4 Fig). Samples from each sensory group were broadly dispersed across the chemistry PCA biplot, revealing minimal alignment between chemical and perceptual dimensions. Terpene contributions to variance explained in the PCA (Table 3) further highlighted the diffuse influence of individual compounds, with ß-myrcene dominating PC1 but having limited relevance to sensory trends observed.

**Fig 7:**
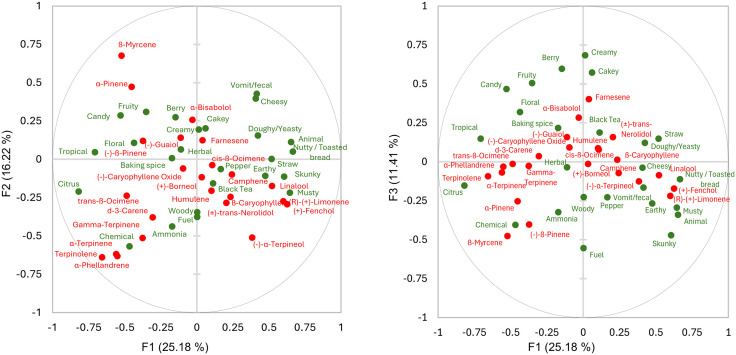
Multi-factor analysis of sensory attributes and terpene chemistry in *Cannabis.*

To probe the potential for integrated chemical-sensory modeling, both MFA (Fig 7) and PLSR (S5 and S6 Figs) were employed. Both models exhibited poor fit, with PLSR in particular showing minimal predictive power. Nevertheless, both MFA and PLSR echoed the isolated significance of terpinolene in relation to *citrus* and *chemical* attributes, as suggested by the univariate and PCA analyses (Fig 7, S4 and S5 Figs). While these techniques often can uncover latent structures in complex datasets, their efficacy was constrained here by sample size limitations and the large number of factors (21 terpenes, 25 sensory; 46 combined). Most statistics literature recommends a minimum sample size between 100 and 250, or a ratio of samples to explanatory factors of at least five to ten for good fit in MFA, which in this case would be 230–460 samples [35]. Similarly for PLSR, the number of samples must be very large with 21 predictive variables. Published guidance for multiple regression analysis suggests approximately 1,200 samples for that number of predictors if the effects are small or just 70 if the effects are large [36].

While the terpene dataset was chemically diverse and statistically discriminable across sensory clusters, it was largely insufficient in accounting for the nuanced and multidimensional nature of sensory perception in *Cannabis*. The scattered correspondence between chemical composition and sensory quality suggests that key perceptual drivers may lie in other, unmeasured compound classes, such as esters, aldehydes, or other volatiles that behave in complex synergistic manners creating characteristic impacts beyond individual terpene contributions.

Similar statistical analyses were performed on the VSC data gathered from type III samples. Type I samples were not analyzed for sulfur compounds due to regulatory constraints of handling high THC samples on a university campus. Interestingly, ANOVA performed on these data did not identify significant differences in individual VSCs or the total VSC content when examined across the four different sensory clusters. PLSR was used to examine relationships between terpenes, VSCs, and sensory attributes in the type III samples (S5 Fig). Similar to the challenges with the terpene data set, the large number of explanatory variables (21 terpenes, 43 VSCs; 64 total) led to an unsatisfactory model fit. Examining measures of fit for the first (and largest) component in the regression output, R^2^ cumulative was 0.145 and Q^2^ cumulative was 0.086, both of which are poor and reflect the same sample size issues faced in MFA. It may also reflect weak correlations among the measured variables or indicate that the current analysis did not include key aroma-contributing compounds, such as certain esters or aldehydes. Future research leveraging broader chemical analyses and larger datasets may be required to uncover more robust predictors of sensory character.

## Discussion

The primary goal of this study was to develop and systematically evaluate a reproducible aroma lexicon for *Cannabis* inflorescence, which is a foundational step toward a standardized, evidence-based framework for describing *Cannabis* aroma. This work did not examine smoke or aerosol aroma but focused exclusively on the uncombusted inflorescence. While this study does not generate or validate a lexicon in a definitive sense, it represents an important phase in the continued evolution of a comprehensive descriptive system for *Cannabis*. It organizes and refines existing sensory terminology into a coherent, data-driven structure suitable for both research and applied contexts. In this way, the lexicon contributes to an ongoing process of refinement and expansion, and its demonstrated ability to consistently differentiate samples underscores its value as a tool for both scientific inquiry and industry communication.

The sensory panel exhibited high reproducibility, supporting the robustness and usability of the lexicon. Although some panelists had limited experience with formal descriptive analysis, minimal training enabled them to produce consistent, high-quality data and accurately characterize blind replicates. This performance suggests that the lexicon is intuitive and accessible, even for those with modest prior sensory experience. Moreover, the use of CATA methodology, which offers substantially higher throughput and lower training demands compared to traditional attribute-scaling techniques, further enhanced the scalability and accessibility of this approach [37,38].

From a sensory standpoint, the lexicon effectively discriminated among samples: all but one descriptor (*baking spice*) significantly differentiated cultivars of both type I and III *Cannabis*, while all but *floral* were effective within type III alone. Even these underperforming attributes approached significance, indicating potential relevance with larger or more diverse sample sets. While 91 samples represent a substantial dataset for lexicon development, further research is needed to capture the full aromatic diversity of *Cannabis*. As part of an iterative process, this lexicon provides a reproducible structure that can be refined and validated through future use, replication, and expansion by other researchers, sensory scientists, and industry professionals.

This study was not initially designed to compare type I and III *Cannabis*. However, results revealed that while both sample types share much of the same aromatic space (which is intuitive considering their classification as the same plant species differing only by THC content, which is a non-volatile compound with no aroma [12]), some distinct sensory differences emerged. Type I samples in this study were more often associated with *skunky*, *musty*, and *animalic* (savory) aromas, while type III samples leaned toward *candy*, *fruity*, *berry*, *cakey*, *citrus*, and *chemical* notes. This divergence likely reflects differences in commonly available genetics within the United States and Europe: type III cultivars typically derive from older, less diverse breeding material, whereas type I breeding has more recently emphasized *musty*, *skunky*, and *gassy* profiles in response to shifting consumer preferences [39].

Across the combined type I and III *Cannabis* samples, four primary aroma profiles appeared to dominate beyond the ubiquitous *herbal* and *woody* aromas. Samples in sensory cluster 1 occupied a sweet profile, with *fruity*, *berry*, and *candy* attributes. Sensory cluster 2 had a *citrus* and *chemical* profile. Sensory cluster 3 had a *cheesy* and *vomit/fecal* profile. These three sensory clusters were comprised mostly of type III samples and to a lesser degree type I samples. Sensory cluster 4 on the other hand was predominantly type I was defined by *skunky, earthy*, *musty, straw, fuel, black tea, woody* and *nutty/toasted bread*. This last group may represent a cluster of distinct aroma families that could be further subdivided in future research.

Two descriptors, *herbal* and *woody*, were frequently used across all or most samples and as a result did not effectively differentiate them. This suggests a need to subdivide these descriptors into two or more specific subcategories. As the data collection progressed, panelists noted perceptible differences within *herbal*, for instance, culinary herbs like *oregano* and *sage*, camphorous notes like *menthol* and *eucalyptus*, and *fresh-cut grass*, and within *woody* panelists commented about different *pine* and *sandalwood* aromas. Breaking these two descriptors into more specific subcategories could improve sample discrimination in the future. Similarly, combining rarely used but similar descriptors like *cakey* and *doughy* could simplify the lexicon without sacrificing nuance. That said, limited representation of certain aroma types in the curated type I sample set may also have suppressed the frequency of terms like *cakey*, suggesting further evaluation across a broader range of *Cannabis* cultivars is warranted. Additionally, while this study contained a large, robust sample set, there are many landrace varieties and preparations of *Cannabis* that could not be included in the study. This study offers a standardized framework for future research, but it is not yet globally representative or a fully complete lexicon. Ongoing efforts should aim to expand the descriptor set to encompass the broad aromatic variation found across *Cannabis* cultivars and growing regions worldwide.

From a chemical standpoint, the study’s terpene data aligned closely with previous findings by Smith et al., who identified six key terpenes, terpinolene, ß-myrcene, ß-caryophyllene, limonene, α-humulene, and linalool, as accounting for most cultivar-level variation [40]. They further suggested three clusters for terpene profiles: caryophyllene-limonene, myrcene-pinene, and terpinolene-myrcene. This finding was largely supported by our findings. For example, clusters with the highest caryophyllene concentrations also contained the most limonene; those high in myrcene also had elevated α-pinene and ß-pinene; and the cluster second-highest in myrcene also had the most terpinolene. Interestingly, α-pinene and farnesene also emerged as potentially important discriminators in our study (Fig 3), though their prominence may reflect sample-specific variation due to our smaller dataset.

VSCs have previously been identified as key aroma molecules, contributing a wide range of sensory notes from savory and skunky to tropical and fruity [13,14]. Although our findings did not replicate the specific results reported by Oswald et al. 2021 or 2023, they also do not contradict them. Importantly, the emphasis of previous studies was on type I *Cannabis* ice hash rosin, which differs substantially from type III inflorescence. Moreover, the present study assessed exclusively type III samples for VSCs. It is plausible that analysis of type I samples, particularly those with high attribute instances of savory and skunky aromas, would have yielded more conclusive results.

While the relationship between terpinolene and *citrus* and *chemical* remained important in the PLSR results of type III samples, the contribution of VSCs was less apparent (S5 Fig). While they were distributed throughout the biplot, there appeared to be a group of VSCs associated with *tropical* and another group associated with *skunky*. However as aforementioned, the model fit was poor and these results should be considered speculative at best.

The lack of fit observed in the model is likely attributable to two main factors: an insufficient sample size and the potential absence of key impact aroma compounds in the *Cannabis* samples analyzed. Other unmeasured compounds, such as esters or aldehydes, may contribute to the aroma profiles of *Cannabis* and represent “unknown unknowns” not captured in this study [15,41]. Incorporating such compounds in future analyses could enhance the explanatory power of PLSR models. Expanding future studies to include a broader range of volatile compounds, as well as both increased number and aromatic breadth of *Cannabis* samples, will be critical for identifying the sensory drivers of *Cannabis* inflorescence. It is also important to consider that while individual compounds may elicit distinct aromas in isolation, their perceptual effects can be altered through interactions with other volatiles. Odorant mixtures are known to generate emergent aromas not predictable from the properties of the individual components, due to complex interactions at peripheral olfactory receptors [34,42].

Despite the chemical groupings, sensory and terpene clustering did not correlate well which underscores a key point: terpene chemistry alone does not predict a sample’s sensory profile. This is crucial given the overreliance on terpene content in current *Cannabis* industry marketing and labeling practices. Total terpene concentration was also not correlated with the frequency of use of each attribute per sample, suggesting that intensity of aroma, which is approximately represented by CATA frequency [29], cannot be directly inferred from total terpene content. Importantly, this study demonstrates that although type I and III *Cannabis* can be somewhat delineated on the basis of their terpene composition, they are aromatically similar. That is, quantitative chemical differences do not translate to perceptual sensory differences.

Nevertheless, certain relationships between specific terpenes and aroma descriptors did emerge. Samples high in terpinolene tended to also receive more *citrus* and *chemical* descriptions even though these two descriptive terms were not used similarly. Cluster analysis of the sensory terms revealed that *citrus* and *chemical* were not clustered together, suggesting they did not lie in the same perceptual dimension and therefore likely reflect different underlying aromas (S7 Fig). While these results are correlative and not causal, they suggest potential associations worth investigating further. Interestingly, d-limonene, widely believed to contribute *citrus* aromas [30–33], was not associated with *citrus* in this study, challenging common assumptions and market narratives.

Although multifactor analysis (MFA) did not explain a large proportion of the overall variance, it provided additional evidence supporting a relationship between terpinolene and both *citrus* and *chemical* sensory attributes. The strong correlation observed between *citrus* and *chemical* notes in both the MFA and PLSR analyses may reflect the dual sensory nature of certain volatile compounds not measured in this study, such as decanal, which has been previously identified in *Cannabis* [16]. Decanal is known to contribute to *citrus* top-notes while also exhibiting aldehydic, waxy characteristics that may underlie the perception of *chemical* nuances.

These findings are especially relevant considering the current *Cannabis* market, which is saturated with inaccurate potency claims [2,43], misleading “effect” descriptions, and persistent safety concerns, including pesticide contamination [44]. As the industry transitions from unregulated to legal frameworks, it’s critical to offer consumers tools for assessing product quality beyond THC content, which has been shown to correlate weakly or negatively with enjoyment [8], and does not impact aroma [12]. In fact, aroma is the only known predictor of subjective enjoyment. However, as this study and others show, terpene profiles do not map cleanly onto sensory attributes [13,15], and traditional classifications such as ‘indica’ and ‘sativa’ have been shown to be unreliable predictors of *Cannabis* attributes [40,45].

This lexicon and its accompanying CATA method provide a high-throughput, low-barrier tool for advancing aroma research in *Cannabis*. By providing a framework for standardized communication, discussions around *Cannabis* aroma can be more informed and effective in the future. Its application can support better cultivar classification, improve product development and marketing strategies, and foster a more transparent, consumer-informed marketplace.

## Conclusions

In summary, this study developed and systematically evaluated a 25-term lexicon for the aroma of *Cannabis* inflorescence. This work lays a foundation for future work to build upon with an initial list of descriptors and an associated sensory method. Future research should incorporate a slightly modified lexicon, for instance with *culinary herbs*, *camphorous*, *grassy*, *pine*, and *sandalwood* descriptors in place of *herbal* and *woody*. Future work requires a larger inflorescence sample size, particularly for defining associations between volatile aromatics and aroma descriptors, and should explore expansion of the lexicon as more terms can be evaluated and validated. The inclusion of consumer hedonic data would deepen our understanding of aroma-driven quality perceptions and consumer liking. Additionally, agronomic and post-harvest variables, including farm origin, harvest maturity, drying methods, storage, and trimming style warrant exploration through both sensory and chemical lenses. Finally, as the lexicon presented here is further expanded and built upon with a more globally representative sample set the attributes present can become more expansive and representative of the wide array of *Cannabis* aromas. Such insights will help guide breeding efforts and optimize production methods aimed at enhancing the aromatic and experiential quality of *Cannabis* products.

## Supporting information

S1 Table Terpenes tested at Columbia Labs with provided LOQ’s. (PDF)

S2 Table Comparison of attribute frequencies from CATA analysis of type I and type III *Cannabis* samples (N = 21 panelists). Differences within each attribute are calculated as average type III *Cannabis* score minus average type I *Cannabis* score where a positive outcome represents greater frequency of use of the attribute for type III *Cannabis* samples while negative outcomes represent greater frequency for the type I *Cannabis* samples.
(PDF)

S3 Table Agglomerative Hierarchical Cluster centroids of sensory data. (PDF)

S4 Table LS means comparisons of terpene profiles of terpene clusters. (PDF)

S5 Table LS means comparisons of terpene profiles of sensory clusters. (PDF)

S1 Fig Temperature and relative humidity in the MCMEC drying chamber. (TIF)

S2 Fig Agglomerative Hierarchical Clustering of type I and type III *Cannabis* highlighting duplicate varieties with different trimming methods. (TIF)

S3 Fig Correspondence Analysis biplots of type I and type III *Cannabis* sensory data with sensory clusters overlaid. Sensory cluster 1 is represented in green diamonds, sensory cluster 2 is represented in blue circles, sensory cluster 3 is represented in pink squares, sensory cluster 4 is represented in orange triangles, and sensory attributes are represented in red circles.
(TIF)

S4 Fig Principal Component Analysis biplot of terpene chemistry of type I and type III *Cannabis* samples with sensory clusters overlaid. Sensory cluster 1 is represented in orange diamonds, sensory cluster 2 is represented in grey squares, sensory cluster 3 is represented in yellow triangles, sensory cluster 4 is represented in green circles, and terpenes are represented in blue circles.
(TIF)

S5 Fig Results of Partial Least Squares Regression of type III *Cannabis* sensory attributes, VSCs, and terpene chemistry. Data was mean centered and scaled by standard deviation before performing this analysis. Comp1 Q^2^cum = 0.086, R^2^Ycum = 0.145, R^2^Xcum = 0.084.
(TIF)

S6 Fig Results of Partial Least Squares Regression of type I and type III *Cannabis* sensory and chemical attributes. Sensory attributes are shown in blue, terpenes shown in red, and type I and type III *Cannabis* samples shown in green. Comp1 Q^2^cum = 0.067, R^2^Ycum = 0.101, R^2^Xcum = 0.217.
(TIF)

S7 Fig Dendrogram of sensory attributes from Agglomerative Hierarchical Clustering. (TIF)
