# Supplementary material for: Beyond potency: A proposed lexicon for sensory differentiation of Cannabis sativa L. aroma
Source: PLoS One. 2025 Oct 21;20(10):e0335125. doi: 10.1371/journal.pone.0335125 (PMC12539713; doi:10.1371/journal.pone.0335125)
Supplement: S1 Table — (PDF) [file pone.0335125.s001.pdf]

S1 Table: Terpenes tested at Columbia Labs with provided LOQ's

Analyte	LOQ	Unit	Analyte	LOQ	Unit
Nerol	0.019695	% w/w	a-Bisabolol	0.019695	% w/w
(±)-fenchone	0.019695	% w/w	Geraniol	0.019695	% w/w
(+)-Cedrol	0.019695	% w/w	Eucalyptol	0.019695	% w/w
trans-ß-Ocimene	0.01313	% w/w	(±)-cis-Nerolidol	0.019695	% w/w
(±)-trans-Nerolidol	0.019695	% w/w	Geranyl acetate	0.019695	% w/w
a-Terpinene	0.019695	% w/w	Sabinene	0.019695	% w/w
(+)-fenchol	0.019695	% w/w	Humulene	0.019695	% w/w
(-)-ß-Pinene	0.019695	% w/w	a-pinene	0.019695	% w/w
Isoborneol	0.019695	% w/w	(±)-Camphor	0.019695	% w/w
p-Cymene	0.019695	% w/w	farnesene	0.019695	% w/w
(-)-Isopulegol	0.019695	% w/w	gamma-Terpinene	0.019695	% w/w
(+)-Pulegone	0.019695	% w/w	ß-Myrcene	0.019695	% w/w
a-phellandrene	0.019695	% w/w	(-)-caryophyllene oxide	0.019695	% w/w
Menthol	0.019695	% w/w	(-)-a-Terpineol	0.019695	% w/w
ß-Caryophyllene	0.019695	% w/w	cis-ß-Ocimene	0.006565	% w/w
Terpinolene	0.019695	% w/w	a-cedrene	0.019695	% w/w
(R)-(+)-Limonene	0.019695	% w/w	Linalool	0.019695	% w/w
Sabinene hydrate	0.019695	% w/w	(-)-Guaiol	0.019695	% w/w
valencene	0.019695	% w/w	Camphene	0.019695	% w/w
d-3-Carene	0.019695	% w/w	(+)-Borneol	0.019695	% w/w
