# Supplementary material for: Beyond potency: A proposed lexicon for sensory differentiation of Cannabis sativa L. aroma
Source: PLoS One. 2025 Oct 21;20(10):e0335125. doi: 10.1371/journal.pone.0335125 (PMC12539713; doi:10.1371/journal.pone.0335125)
Supplement: S2 Table — Differences within each attribute are calculated as average type III Cannabis score minus average type I Cannabis score where a positive outcome represents greater frequency of use of the attribute for type III Cannabis samples while negative outcomes represent greater frequency for the type I Cannabis samples. (PDF) [file pone.0335125.s002.pdf]

S2 Table: Comparison of attribute frequencies from CATA analysis of type I and type III *Cannabis* samples (N = 21 panelists). Differences within each attribute are calculated as average type III *Cannabis* score minus average type I *Cannabis* score where a positive outcome represents greater frequency of use of the attribute for type III *Cannabis* samples while negative outcomes represent greater frequency for the type I *Cannabis* samples.

| | Type III *Cannabis* | | | | | Type I *Cannabis* | | | | | |
|---|---|---|---|---|---|---|---|---|---|---|---|
| Attribute | Sum | % | Min | Max | Avg | Sum | % | Min | Max | Avg | Difference |
| Herbal | 554 | 100% | 4 | 15 | 9.4 | 252 | 100% | 2 | 12 | 7.9 | 1.5 |
| Citrus | 477 | 86% | 0 | 16 | 8.1 | 185 | 73% | 0 | 14 | 5.8 | 2.3 |
| Woody | 399 | 72% | 1 | 16 | 6.8 | 216 | 86% | 3 | 12 | 6.8 | 0.0 |
| Fruity | 376 | 68% | 0 | 13 | 6.4 | 180 | 71% | 0 | 13 | 5.6 | 0.7 |
| Candy | 366 | 66% | 0 | 14 | 6.2 | 152 | 60% | 0 | 14 | 4.8 | 1.5 |
| Floral | 292 | 53% | 0 | 9 | 4.9 | 124 | 49% | 0 | 9 | 3.9 | 1.1 |
| Straw | 283 | 51% | 1 | 15 | 4.8 | 165 | 65% | 1 | 9 | 5.2 | -0.4 |
| Tropical | 281 | 51% | 0 | 9 | 4.8 | 107 | 42% | 0 | 9 | 3.3 | 1.4 |
| Earthy | 223 | 40% | 1 | 14 | 3.8 | 134 | 53% | 0 | 10 | 4.2 | -0.4 |
| Chemical | 218 | 39% | 0 | 11 | 3.7 | 128 | 51% | 0 | 10 | 4.0 | -0.3 |
| Black Tea | 182 | 33% | 0 | 12 | 3.1 | 104 | 41% | 1 | 7 | 3.3 | -0.2 |
| Cheesy | 166 | 30% | 0 | 11 | 2.8 | 83 | 33% | 0 | 10 | 2.6 | 0.2 |
| Pepper | 159 | 29% | 0 | 9 | 2.7 | 84 | 33% | 0 | 9 | 2.6 | 0.1 |
| Ammonia | 149 | 27% | 0 | 6 | 2.5 | 106 | 42% | 0 | 7 | 3.3 | -0.8 |
| Musty | 145 | 26% | 0 | 10 | 2.5 | 129 | 51% | 0 | 10 | 4.0 | -1.6 |
| Vomit/fecal | 143 | 26% | 0 | 10 | 2.4 | 69 | 27% | 0 | 7 | 2.2 | 0.3 |
| Berry | 140 | 25% | 0 | 8 | 2.4 | 81 | 32% | 0 | 8 | 2.5 | -0.2 |
| Fuel | 134 | 24% | 0 | 7 | 2.3 | 94 | 37% | 0 | 7 | 2.9 | -0.7 |
| Animal | 104 | 19% | 0 | 8 | 1.8 | 79 | 31% | 0 | 9 | 2.5 | -0.7 |
| Skunky | 93 | 17% | 0 | 5 | 1.6 | 98 | 39% | 0 | 10 | 3.1 | -1.5 |
| Nutty / Toasted bread | 87 | 16% | 0 | 7 | 1.5 | 93 | 37% | 0 | 10 | 2.9 | -1.4 |
| Creamy | 79 | 14% | 0 | 7 | 1.3 | 49 | 19% | 0 | 6 | 1.5 | -0.2 |
| Cakey | 78 | 14% | 0 | 7 | 1.3 | 53 | 21% | 0 | 6 | 1.7 | -0.3 |
| Baking spice | 70 | 13% | 0 | 7 | 1.2 | 34 | 13% | 0 | 3 | 1.1 | 0.1 |
| Doughy/Yeasty | 43 | 8% | 0 | 4 | 0.7 | 35 | 14% | 0 | 4 | 1.1 | -0.4 |
