# Supplementary material for: Beyond potency: A proposed lexicon for sensory differentiation of Cannabis sativa L. aroma
Source: PLoS One. 2025 Oct 21;20(10):e0335125. doi: 10.1371/journal.pone.0335125 (PMC12539713; doi:10.1371/journal.pone.0335125)
Supplement: S3 Table — (PDF) [file pone.0335125.s003.pdf]

S3 Table: Agglomerative Hierarchical Cluster centroids of sensory data

Cluster	Fruity	Citrus	Berry	Tropical	Earthy	Musty
1	9.6	4.9	4.8	5.1	3.2	2.2
2	6.3	12.1	2.1	5.7	2.9	1.7
3	5.6	5.7	2.4	3.6	3.9	3.5
4	2.5	2.0	0.5	1.3	6.9	6.0

Cluster	Pepper	Herbal	Floral	Straw	Baking spice	Candy
1	1.8	8.3	5.5	5.6	1.8	9.4
2	2.5	9.1	5.3	3.4	1.1	6.4
3	2.8	8.4	3.9	4.7	0.7	5.1
4	3.8	9.7	3.0	7.8	1.0	0.9

Cluster	Fuel	Chemical	Ammonia	Cheesy	Creamy	Cakey
1	1.3	1.3	1.3	2.0	3.2	2.7
2	3.2	6.3	3.7	1.0	0.9	1.1
3	1.8	2.9	2.3	5.8	1.3	1.5
4	3.4	2.7	3.1	2.9	0.4	0.8

Cluster	Animal	Vomit/fecal	Black Tea	Skunky	Doughy/Yeasty	Woody
1	1.9	2.3	2.8	0.9	0.8	4.9
2	0.6	0.4	3.2	1.1	0.4	6.9
3	3.2	5.2	2.4	2.7	1.3	6.7
4	3.5	2.2	4.5	4.6	1.3	8.5

Cluster	Nutty / Toasted bread
1	1.5
2	0.6
3	2.6
4	4.4
