# Supplementary material for: Beyond potency: A proposed lexicon for sensory differentiation of Cannabis sativa L. aroma
Source: PLoS One. 2025 Oct 21;20(10):e0335125. doi: 10.1371/journal.pone.0335125 (PMC12539713; doi:10.1371/journal.pone.0335125)
Supplement: S4 Table — (PDF) [file pone.0335125.s004.pdf]

S4 Table: LS means comparisons of terpene profiles of terpene clusters

Category	(+)-Fenchol	(-)-Guaiol	(-)-ß-Pinene	Gamma-Terpinene	(-)-α-Terpineol	Linalool	Humulene
A	0.004 b	0.056 a	0.091 a	0.000 b	0.006 c	0.039 b	0.090 b
B	0.006 b	0.058 a	0.079 a	0.010 a	0.028 b	0.029 b	0.107 ab
C	0.056 a	0.013 b	0.073 a	0.000 b	0.058 a	0.260 a	0.148 a
D	0.012 b	0.070 a	0.020 b	0.000 b	0.011 c	0.066 b	0.071 b
Pr > F(Model)	<0.0001	0.014	<0.0001	<0.0001	<0.0001	<0.0001	0.009
Significant	Yes	Yes	Yes	Yes	Yes	Yes	Yes

Category	α-Pinene	Terpinolene	(-)-Caryophyllene Oxide	ß-Myrcene	α-Bisabolol	ß-Caryophyllene	(±)-trans-Nerolidol
A	0.265 a	0.100 b	0.006 a	0.900 a	0.082 a	0.226 b	0.012 b
B	0.108 b	0.847 a	0.009 a	0.434 b	0.052 a	0.258 b	0.024 ab
C	0.047 b	0.003 b	0.006 a	0.215 c	0.042 a	0.404 a	0.038 a
D	0.036 b	0.017 b	0.005 a	0.165 c	0.083 a	0.191 b	0.028 ab
Pr > F(Model)	<0.0001	<0.0001	0.714	<0.0001	0.034	0.000	0.008
Significant	Yes	Yes	No	Yes	Yes	Yes	Yes

Category	(+)-Borneol	d-3-Carene	(R)-(+)-Limonene	Farnesene	α-Terpinene	trans-ß-Ocimene	cis-ß-Ocimene
A	0.000 a	0.003 b	0.151 b	0.141 ab	0.001 b	0.043 b	0.000 a
B	0.000 a	0.024 a	0.169 b	0.063 b	0.029 a	0.129 a	0.000 a
C	0.001 a	0.000 b	0.774 a	0.064 b	0.000 b	0.010 b	0.000 a
D	0.000 a	0.000 b	0.148 b	0.220 a	0.000 b	0.027 b	0.000 a
Pr > F(Model)	0.258	<0.0001	<0.0001	0.024	<0.0001	<0.0001	0.258
Significant	No	Yes	Yes	Yes	Yes	Yes	No

Category	Camphene	α-Phellandrene
A	0.000 a	0.001 b
B	0.000 a	0.032 a
C	0.002 a	0.000 b
D	0.000 a	0.000 b
Pr > F(Model)	0.039	<0.0001
Significant	Yes	Yes

P-values correspond to F-values from univariate ANOVA tests evaluating the effect of sensory category treatment. Means that do not share the same “a”, “b”, or “c” grouping are significantly different at a p<0.05 based on a Least Square Means comparisons.
