# Supplementary material for: Beyond potency: A proposed lexicon for sensory differentiation of Cannabis sativa L. aroma
Source: PLoS One. 2025 Oct 21;20(10):e0335125. doi: 10.1371/journal.pone.0335125 (PMC12539713; doi:10.1371/journal.pone.0335125)
Supplement: S5 Table — (PDF) [file pone.0335125.s005.pdf]

S5 Table: LS means comparisons of terpene profiles of sensory clusters

Category	(+)-Fenchol	(-)-Guaiol	(-)-ß-Pinene	Gamma-Terpinene	(-)-α-Terpineol	Linalool	Humulene
1	0.014 b	0.052 a	0.070 ab	0.000 a	0.018 a	0.056 a	0.114 a
2	0.008 b	0.044 a	0.091 a	0.004 a	0.020 a	0.057 a	0.098 a
3	0.016 b	0.071 a	0.062 b	0.001 a	0.018 a	0.109 a	0.077 a
4	0.033 a	0.032 a	0.053 b	0.000 a	0.031 a	0.143 a	0.131 a
Pr > F(Model)	0.008	0.155	0.005	0.034	0.366	0.055	0.120
Significant	Yes	No	Yes	Yes	No	No	No

Category	α-Pinene	Terpinolene	(-)-Caryophyllene Oxide	ß-Myrcene	α-Bisabolol	ß-Caryophyllene	(±)-trans-Nerolidol
1	0.152 a	0.040 b	0.006 a	0.578 a	0.093 a	0.275 ab	0.018 a
2	0.204 a	0.498 a	0.007 a	0.635 a	0.059 a	0.240 b	0.023 a
3	0.144 a	0.064 b	0.006 a	0.584 a	0.063 a	0.208 b	0.021 a
4	0.044 b	0.023 b	0.007 a	0.251 b	0.068 a	0.370 a	0.028 a
Pr > F(Model)	0.004	<0.0001	0.949	0.010	0.240	0.019	0.788
Significant	Yes	Yes	No	Yes	No	Yes	No

Category	α-Phellandrene	d-3-Carene	(R)-(+)-Limonene	Farnesene	α-Terpinene	trans-ß-Ocimene	cis-ß-Ocimene
1	0.002 b	0.002 a	0.283 b	0.189 a	0.001 b	0.028 b	0.000 a
2	0.015 a	0.011 a	0.185 b	0.090 a	0.014 a	0.087 a	0.000 a
3	0.002 b	0.007 a	0.252 b	0.111 a	0.002 b	0.046 b	0.000 a
4	0.000 b	0.000 a	0.504 a	0.143 a	0.000 b	0.006 b	0.000 a
Pr > F(Model)	<0.0001	0.096	0.004	0.267	<0.0001	0.000	0.258
Significant	Yes	No	Yes	No	Yes	Yes	No

Category	Camphene	(+)-Borneol
1	0.001 a	0.000 a
2	0.000 a	0.001 a
3	0.000 a	0.000 a
4	0.001 a	0.000 a
Pr > F(Model)	0.339	0.649
Significant	No	No

P-values correspond to F-values from univariate ANOVA tests evaluating the effect of sensory category treatment. Means that do not share the same “a” or “b” grouping are significantly different at a p<0.05 based on a Least Square Means comparisons.
