# Supplementary figures and images for: Beyond potency: A proposed lexicon for sensory differentiation of Cannabis sativa L. aroma
Source: PLoS One. 2025 Oct 21;20(10):e0335125. doi: 10.1371/journal.pone.0335125 (PMC12539713; doi:10.1371/journal.pone.0335125)

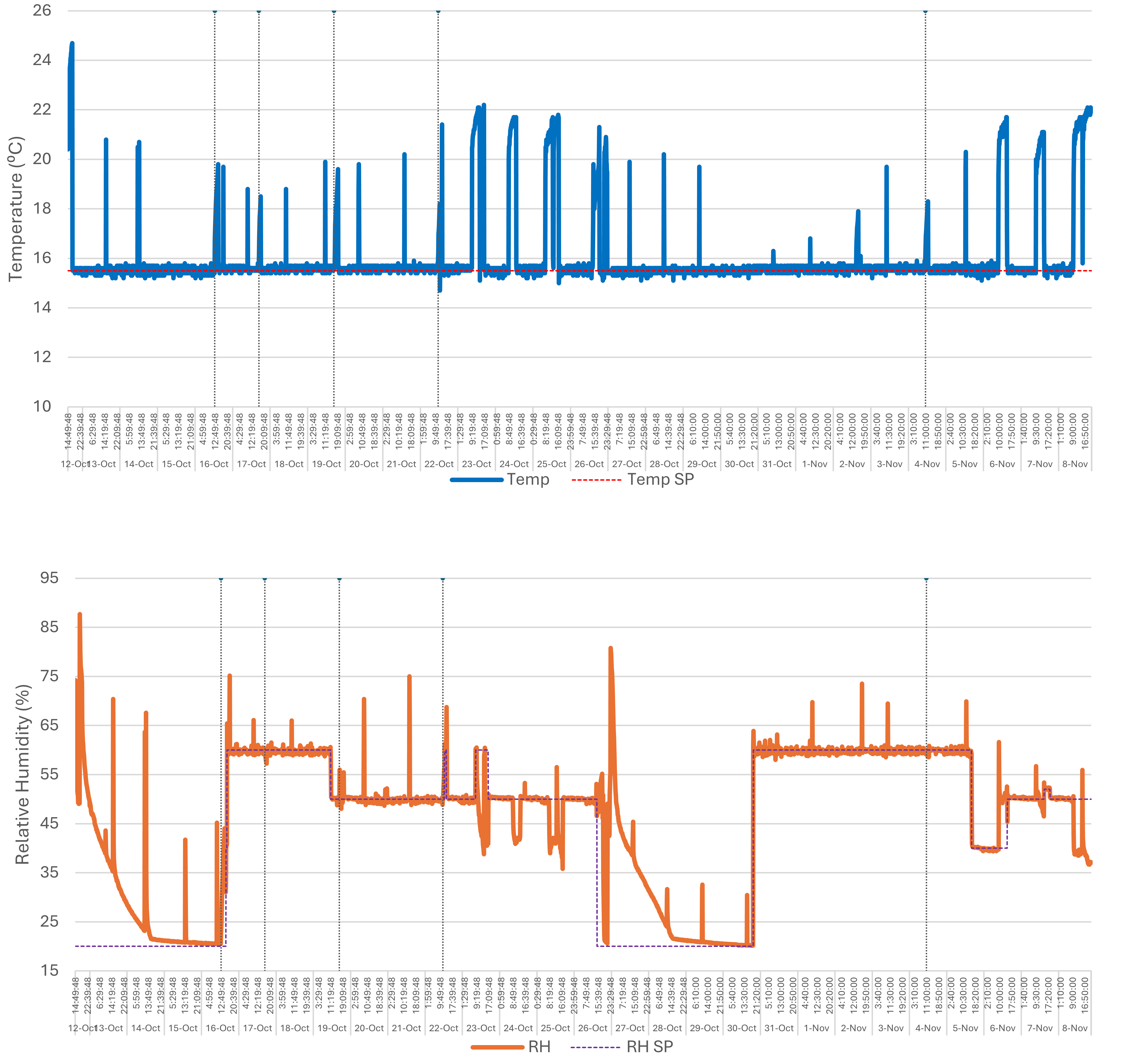

Supplement: S1 Fig — (TIF) [file pone.0335125.s006.tif]

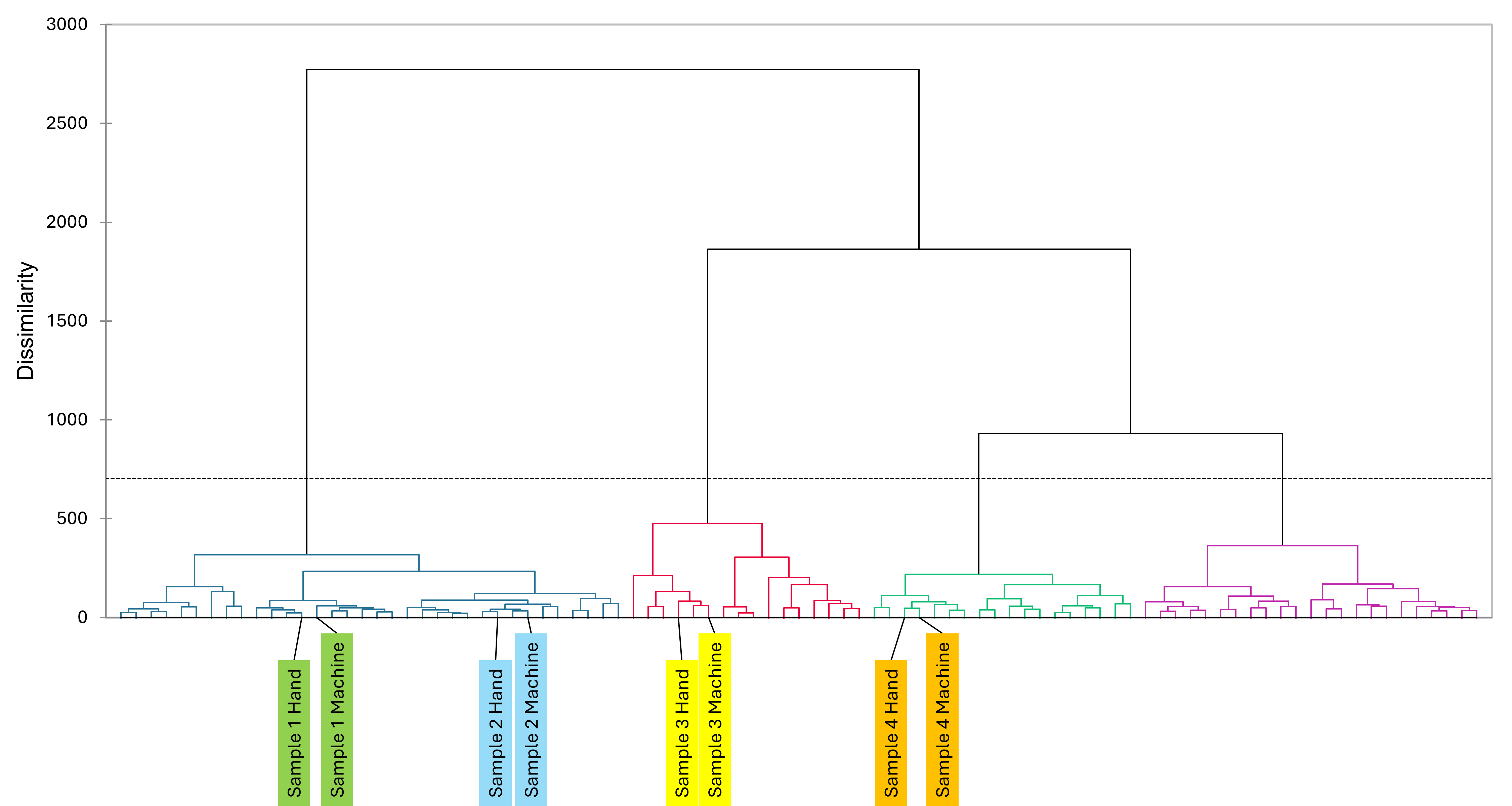

Supplement: S2 Fig — (TIF) [file pone.0335125.s007.tif]

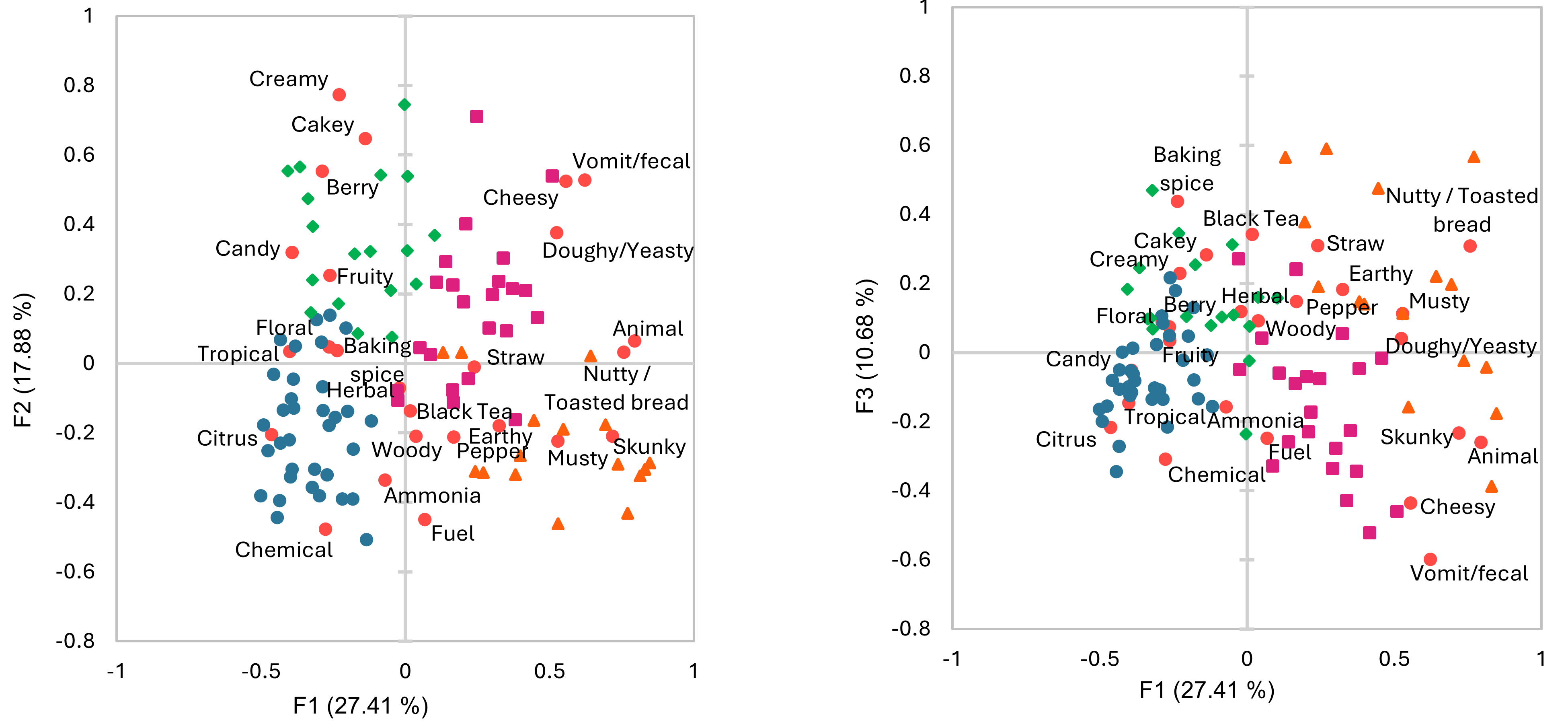

Supplement: S3 Fig — Sensory cluster 1 is represented in green diamonds, sensory cluster 2 is represented in blue circles, sensory cluster 3 is represented in pink squares, sensory cluster 4 is represented in orange triangles, and sensory attributes are represented in red circles. (TIF) [file pone.0335125.s008.tif]

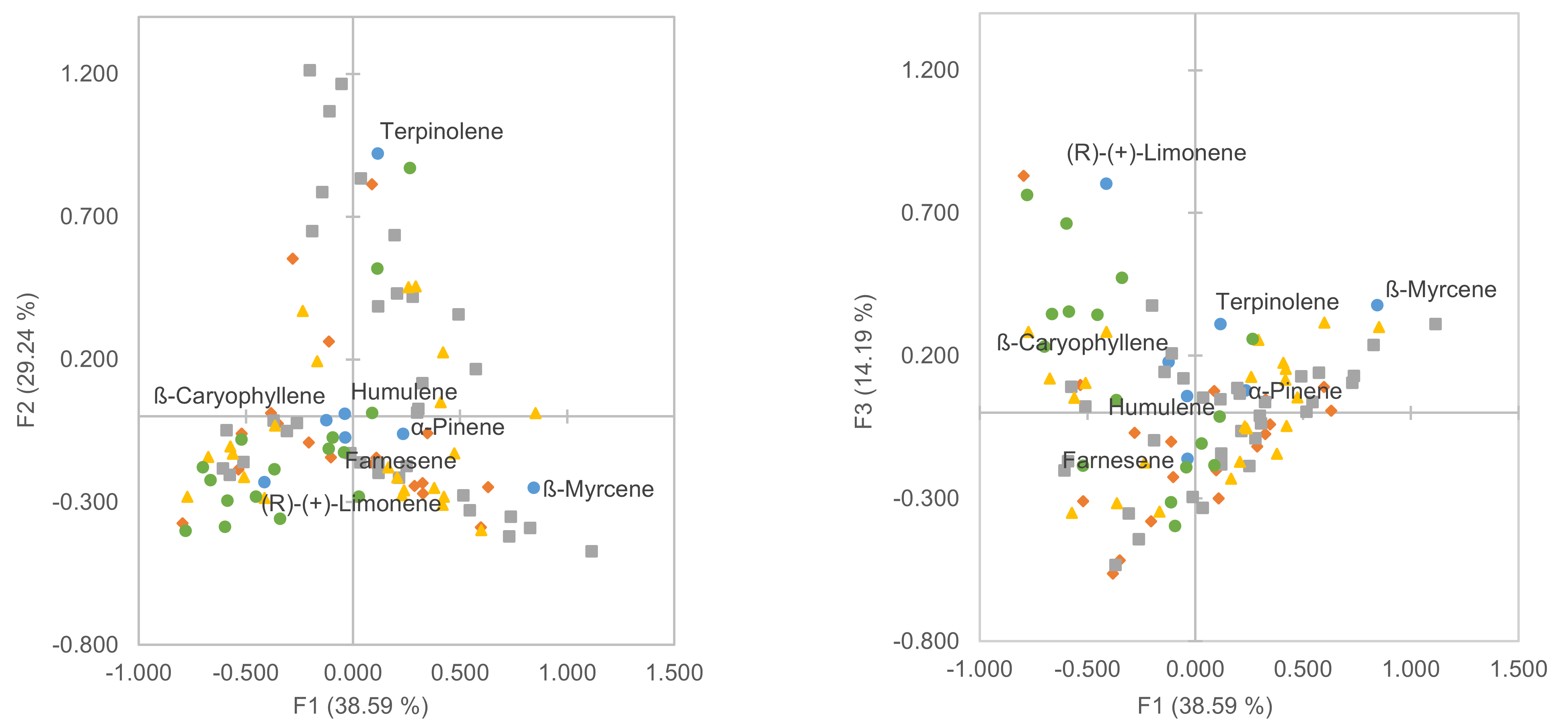

Supplement: S4 Fig — Sensory cluster 1 is represented in orange diamonds, sensory cluster 2 is represented in grey squares, sensory cluster 3 is represented in yellow triangles, sensory cluster 4 is represented in green circles, and terpenes are represented in blue circles. (TIF) [file pone.0335125.s009.tif]

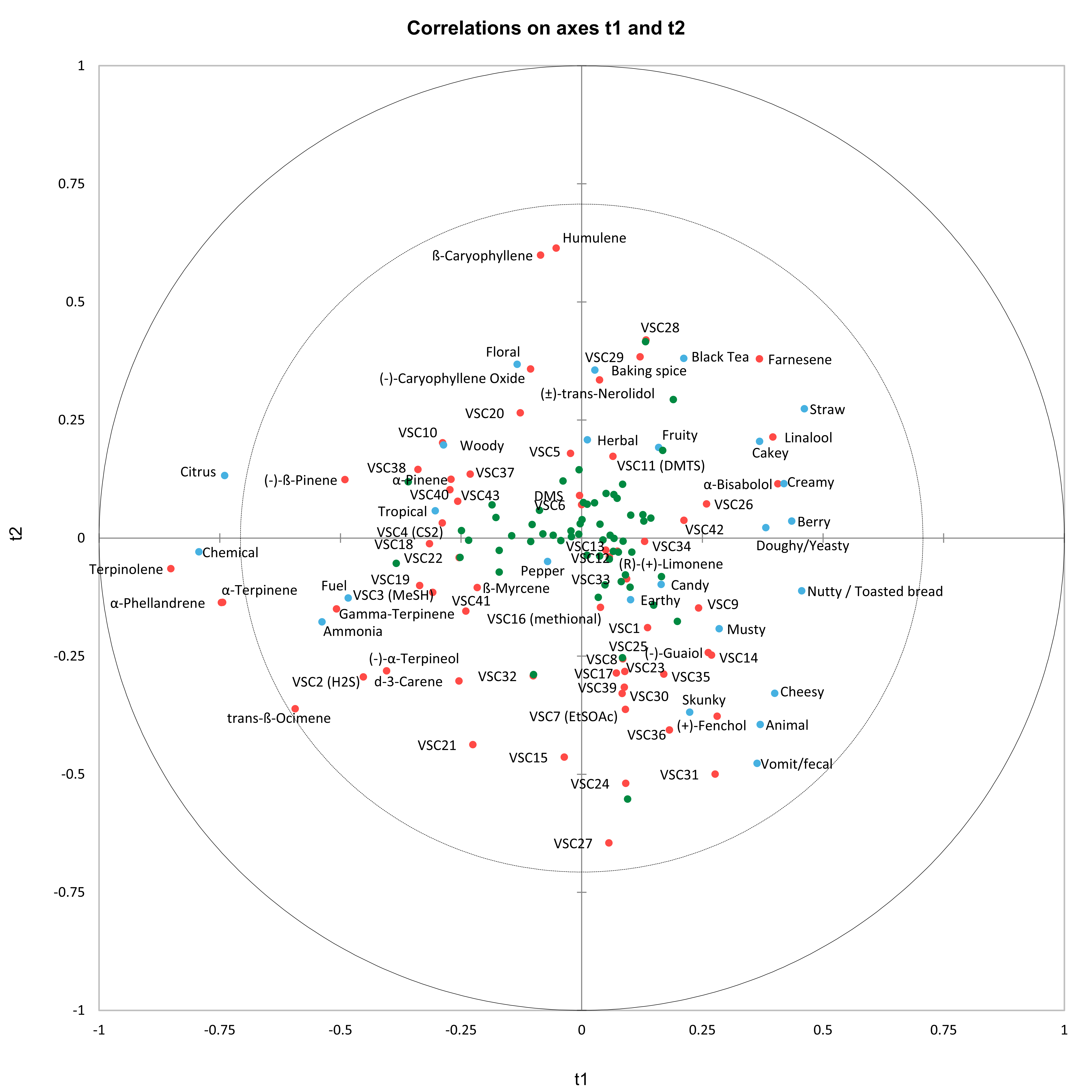

Supplement: S5 Fig — Data was mean centered and scaled by standard deviation before performing this analysis. Comp1 Q2cum = 0.086, R2Ycum = 0.145, R2Xcum = 0.084. (TIF) [file pone.0335125.s010.TIF]

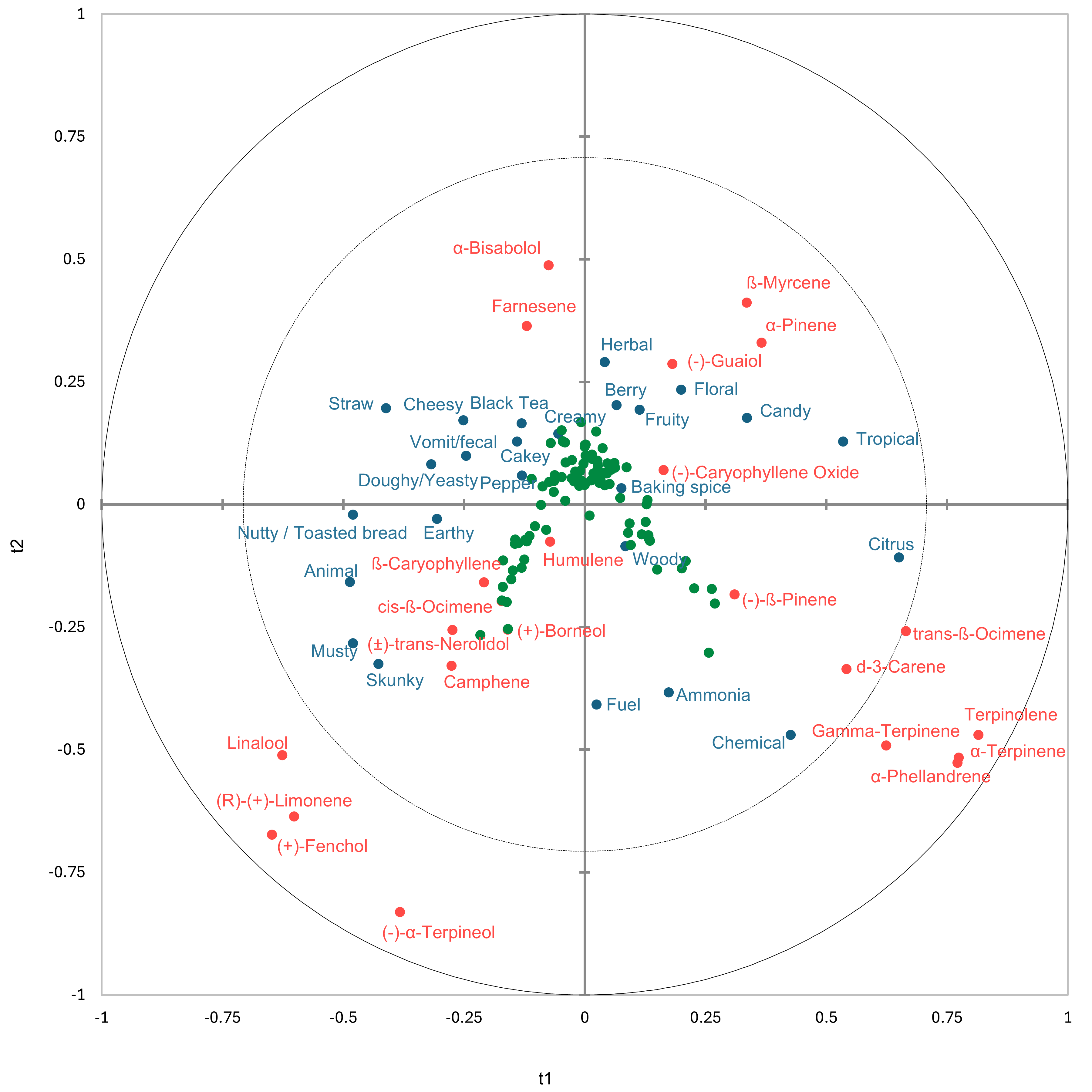

Supplement: S6 Fig — Sensory attributes are shown in blue, terpenes shown in red, and type I and type III Cannabis samples shown in green. Comp1 Q2cum = 0.067, R2Ycum = 0.101, R2Xcum = 0.217. (TIF) [file pone.0335125.s011.tif]

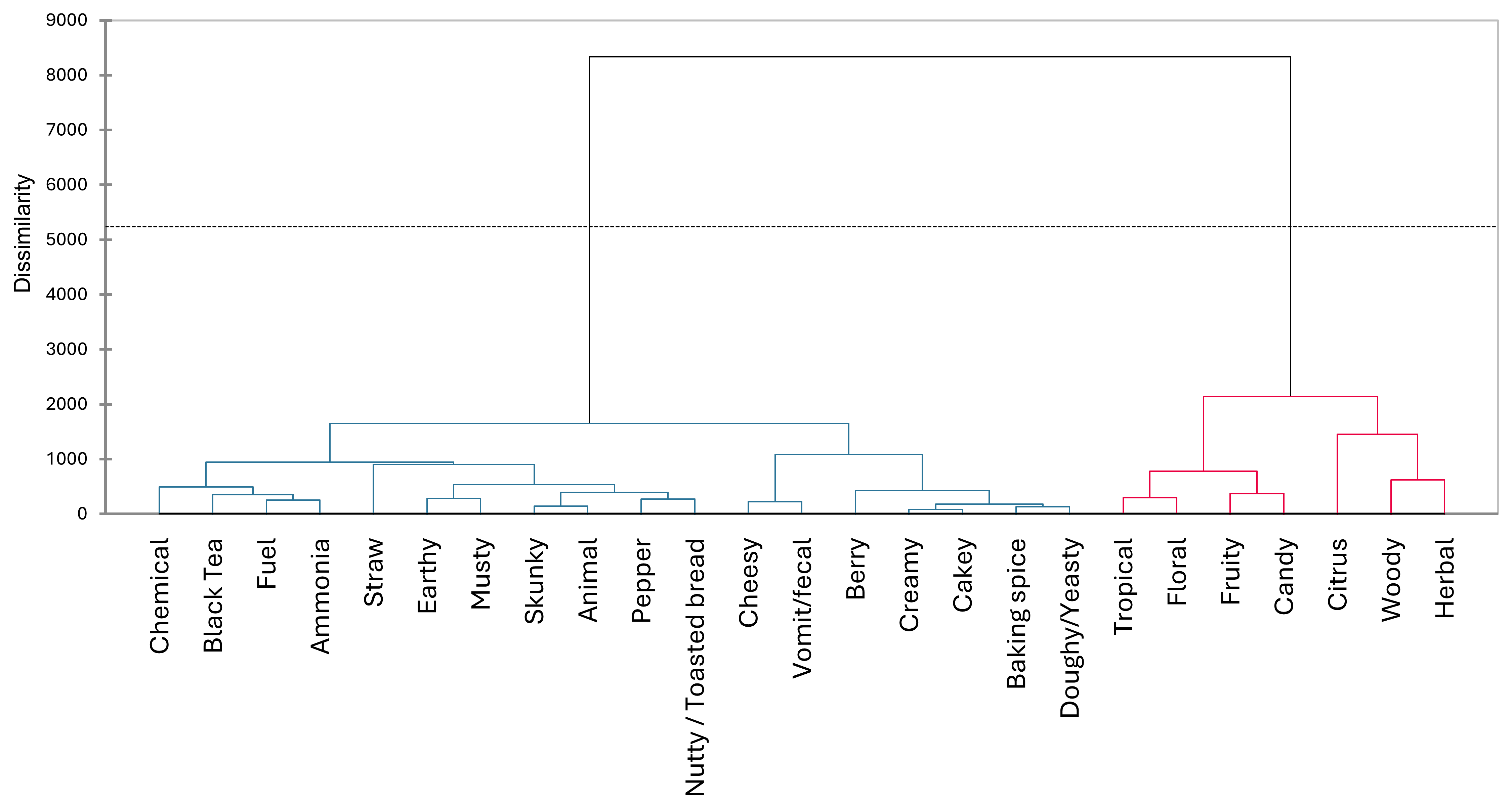

Supplement: S7 Fig — (TIF) [file pone.0335125.s012.tif]
